# Emergency Department Disposition and Point-of-Care Ultrasound in Biliary Disease: Propensity-Weighted Cohort Study

**DOI:** 10.5811/westjem.47347

**Published:** 2025-11-26

**Authors:** Yamato Eda, Po-sheng Wu, Fen-Wei Huang, Sheng-Yao Hung, Ching-Ting Hsu, Wei-Kung Chen, Shih-Hao Wu

**Affiliations:** * China Medical University Hospital, Department of Emergency Medicine, Taichung, Taiwan; † China Medical University, College of Medicine, School of Medicine, Taichung, Taiwan

## Abstract

**Introduction:**

Biliary tract disease is a frequent cause of abdominal pain among emergency department (ED) patients and accounts for a significant portion of hospital admissions and return visits. Our objective was to compare ED outcomes for patients ultimately diagnosed with biliary tract disease based on the use of point-of-care ultrasound (POCUS) during their initial visit. We specifically analyzed patients admitted after an unscheduled return visit within 72 hours vs those admitted directly from the ED.

**Methods:**

In this retrospective cohort study we used propensity score weighting and included 1,228 adults admitted for biliary tract disease, either during their initial ED visit (n = 1,120, 91.2%) or following an unscheduled return visit within 72 hours (n = 108, 8.8%) at a tertiary center in Taiwan between 2021–2023. Outcomes included ED length of stay (LOS), costs, hospital LOS, intensive care unit (ICU) admission, and in-hospital mortality. We used multivariable regression models with inverse probability of treatment weighting adjustment to account for baseline differences.

**Results:**

Initial discharge followed by admission after an unscheduled return visit was not associated with worse clinical outcomes compared to direct admission. There were no significant differences in in-hospital mortality (0.93% vs 1.16%; odds ratio [OR] 0.59, *P* = .56) or ICU admission (0.93% vs 0.71%; OR 1.78, *P* = .61). While the initial ED LOS was shorter (mean: 4 hours vs 15.6 hours; regression-adjusted difference −6.66 hours, *P* < .001) and the initial ED costs were lower (mean: NT5477 vs NT$16,269, a 66% savings; regression-adjusted difference: −NT$6,548, *P* < .001), this reflects an expected early discharge. Among patients ultimately requiring admission after an unscheduled return visit, those who received POCUS at their index visit had a significantly shorter initial ED LOS (mean: 2.97 hours vs 4.78 hours; regression-adjusted difference −1.42 hours, *P* = .006) and lower initial ED costs (mean: NT$3,248 vs NT$7,149; a 55% saving; regression-adjusted difference −NT$3,271, *P* < .001) compared to those who did not. This initial POCUS use did not increase adverse events; only one of the 108 patients in the unscheduled return visit group required ICU admission (0.9%, 95% CI, 0.02–5.1%), and no deaths occurred (0%, 95% CI, 0–2.78%).

**Conclusion:**

Initial discharge following ED assessment appears safe for many low-risk patients ultimately diagnosed with biliary tract disease on repeat visit within 72 hours. Incorporating POCUS during the initial evaluation may shorten ED LOS and reduce costs for patients who later require admission, without apparent measurable negative effects on mortality, hospital, or ICU length of stay.

## INTRODUCTION

Biliary tract disease is a frequent cause of abdominal pain among emergency department (ED) patients and accounts for a significant portion of hospital admissions and return visits.^1^ Delayed or missed diagnoses can lead to serious complications, including sepsis, biliary obstruction, and organ failure.^2^–^4^ Consequently, there is a critical need for diagnostic strategies that support timely identification and management of biliary tract disease in the ED.

Point-of-care ultrasound (POCUS), often described as the “ultrasound stethoscope,” has become an essential bedside tool for emergency physicians.^5^ Prior research has demonstrated its effectiveness in enhancing diagnostic accuracy, expediting care, and reducing ED length of stay (LOS) in various conditions such as renal colic, soft tissue infections, and bowel obstruction.^6^, ^7^ In biliary tract disease specifically, POCUS has shown high sensitivity and specificity, particularly for detecting cholelithiasis,^8^ and enables clinicians to rapidly identify pathology, potentially reducing reliance on computed tomography (CT) and other resource-intensive imaging modalities.^9^

Efficiency in the ED is a key determinant of patient safety and overall system performance.^10^ Crowding is linked to delays in care, increased healthcare costs, and adverse outcomes, including in-hospital cardiac arrest.^11^, ^12^ Thus, reducing ED LOS and optimizing throughput are high priorities.^12^ Point-of-care ultrasound may serve as a frontline imaging strategy that facilitates faster decision-making, lowers costs, and improves patient flow,^13^ especially when compared to CT, which may be less readily available or involve longer wait times.^9^, ^14^, ^15^

Unscheduled return visits within 72 hours are commonly used as a quality-of-care metric and are often viewed as indicators of premature discharge, diagnostic error, or suboptimal treatment.^16^, ^17^ While many unscheduled return visits are unavoidable, a substantial proportion are preventable,^18^–^21^ and can contribute to ED crowding and increased healthcare expenditures.^22^, ^23^ In the context of biliary tract disease, patients discharged from the ED may experience progression of disease, requiring admission upon return.^24^, ^25^ Comparing outcomes between patients admitted directly and those admitted following an unscheduled return visit can offer insights into whether early discharge, particularly after POCUS-based evaluation, compromises patient safety.^26^

Although existing studies support the diagnostic accuracy and cost effectiveness of POCUS for biliary tract disease,^15^, ^27^ limited evidence exists on its role in guiding safe discharge decisions. Specifically, the association between POCUS and patient outcomes for those discharged after the initial visit but later readmitted has not been well characterized.

We hypothesized that POCUS-guided discharge decisions do not adversely affect patient outcomes and may contribute to more efficient ED resource utilization in patients ultimately diagnosed with biliary tract disease. In this study we aimed to evaluate the association of POCUS at the initial ED visit with ED LOS, medical costs, and safety outcomes in patients admitted for biliary tract disease after an unscheduled return visit, compared to those admitted directly at their initial ED presentation.

**Population Health Research Capsule**

What do we already know about this issue?

*ED discharge decisions for patients with nonspecific abdominal pain are complex.*

What was the research question?

*What is the association of initial ED point-of-care ultrasound (POCUS) with outcomes for biliary tract disease patients admitted directly or after revisit?*

What was the major finding of the study?

*In admissions of patients with biliary tract disease after an unscheduled return visit, initial POCUS use had a shorter ED LOS (−1.42 hours, P = .006) and lower costs by 55%.*

How does this improve population health?

*Integrating POCUS into ED assessment enhances efficiency, reduces costs, and allows safe discharge for low-risk patients with biliary tract disease.*

## METHODS

### Study Design

This retrospective cohort study used propensity score weighting with data from the electronic health record (EHR) of a tertiary medical center in Taiwan with more than 160,000 ED visits annually, between January 1, 2021–December 31, 2023. The database is de-identified but contains a unique, encrypted personal identifier that allows researchers to link claims between ED and inpatient databases. This study was approved by the Institutional Review Board of China Medical University Hospital (CMUH113-REC2-008), and informed consent was waived. We followed STROBE guidelines for observational studies, and all elements in the checklist for cross-sectional studies are presented in content and structure.^28^

All data were initially extracted by the staff in the hospital’s information department according to our application form. Further data were extracted by a trained research assistant (RA), following 12 recommended criteria for medical record review studies.^29^ The RA was blinded to the study hypotheses and had a 30-minute training session on the ED medical record abstraction process. According to the inclusion criteria and definition of subgroups, the number of cases extracted by RA was compared with that of the researchers through data selection in Excel (Microsoft Corporation, Redmond, WA). There was 100% interobserver reliability of the variables of interest for this cohort.

### Population and Setting

This retrospective study was based on a larger group of adults (≥ 18 years of age) who presented at the ED with non-traumatic abdominal pain. We then selected those who were diagnosed with biliary tract disease (*International Classification of Diseases, 10^th^ Revision* [*ICD-10*] codes K80–K83) either at their initial hospital admission or admission after an unscheduled return visit within 72 hours, regardless of whether they were pregnant or not. Patients were excluded if they were < 18 years of age, had traumatic abdominal pain, were transferred from other EDs, or had been discharged against medical advice from other clinics or hospitals.

We characterized patients into two groups: 1) those discharged after the index ED visit but subsequently admitted with a biliary tract disease diagnosis upon an unscheduled return visit within 72 hours (n = 108); and 2) those admitted directly during the index visit with a biliary tract disease diagnosis (n = 1,120). In total, 1,228 patients were included in the core analysis.

All physicians performing POCUS had completed mandatory residency training, standard in Taiwan since 2012, which requires a standardized hands-on assessment and regular refresher courses under certified instructors, and are responsible for performing and interpreting all examinations. There has been no radiology ultrasonography available in our ED for at least seven years.

In this study, POCUS was not limited to evaluating a specific organ or confirming a diagnosis. Instead, it was used to address the clinical questions that arise during patient care, often requiring a comprehensive assessment across multiple organs and systems based on the patient’s presenting symptoms. All physicians at this tertiary center had received standardized POCUS training and could apply any protocol or technique they had learned to guide their clinical decisions. Take the example of biliary POCUS. It must include longitudinal and transverse views of the gallbladder, including the gallbladder neck, with or without the echo-Murphy sign, pericholecystic edema. It should also include a measurement of the thickness of the gallbladder wall and an assessment of the common bile duct.^30^ We examined the association between POCUS and admission, ED LOS, and related medical costs after an unscheduled return visit, by comparing patients who underwent POCUS at the index visit with those who did not.

We defined an index visit as an ED visit without a prior ED visit or hospitalization in the preceding 72 hours. A return visit was defined as any ED revisit within 72 hours of discharge; for patients with multiple revisits, only the first was included. The unit of analysis was the visit; a single patient could contribute multiple index visits during the study period. We focused on early revisits (within 72 hours), as these cases are generally more preventable and more responsive to ED- or hospital-based quality improvement interventions.^31^

To assess the role of POCUS in patient safety, we compared patients in the POCUS-only group at the index visit who required admission after an unscheduled return visit with patients in the CT group admitted directly at the index visit. Furthermore, to examine the cost and efficiency of POCUS, we compared patients in the POCUS-only group at the index visit who required admission after an unscheduled return visit with patients who received neither POCUS nor CT at the index visit who required admission after an unscheduled return visit.

### Variables

The EHR contains information on patient demographics, visit date and time, triage level, comorbidities, *ICD-10* diagnostic codes, POCUS, CT, ED disposition, ED LOS, costs, and hospital LOS. The Taiwan Triage and Acuity Scales system is a computerized, five-level system with acuity levels 1–5 indicating resuscitation, emergent, urgent, less urgent, and non-urgent, respectively.^32^

### Outcome Measures

The outcome measures were ED LOS, ED costs, inpatient mortality, intensive care unit (ICU) admission, hospital LOS, and total ED and inpatient costs in New Taiwan dollars (NT$). The ED LOS was defined as the period from the patient’s initial presentation to the ED, as documented by the triage nurse, to the patient’s discharge from the ED. We calculated ED LOS at the following five points: discharge from the ED; discharge from the observation room; admission to the general ward; admission to the ICU; and ED mortality. The hospital LOS of patients admitted to the general ward or ICU was documented as a secondary outcome to evaluate the prognosis of patients.

### Data Analysis

Summary statistics are presented as means (with standard deviations). We examined bivariate associations using the Student *t*-test, and chi-square tests, as appropriate. We analyzed the clinical outcomes (mortality and ICU admission) and resource use (LOS and cost) by comparing the patients admitted for biliary tract disease after an unscheduled return visit to those admitted directly after the index visit, and the POCUS-only group admitted after an unscheduled return visit to the CT-only group in patients who were admitted directly after the index visit, and to patients who did not receive POCUS at the index visit who required admission after an unscheduled return visit as a sensitivity analysis. Only seven missing data points were found, all of which were limited to vital signs and anthropometric measures (ie, weight, height, and body mass index [BMI]). We excluded these data points from the calculations of means and standard deviations.

To account for differences in baseline characteristics, we employed multivariable logistic and linear regression models, adjusting for potential confounders including age, sex, triage level, BMI, and comorbidities. To further reduce confounding bias in group comparisons, we applied inverse probability of treatment weighting (IPTW) based on propensity scores derived from a logistic regression model using the aforementioned covariates. Stabilized weights were used to minimize the influence of extreme propensity scores. We evaluated covariate balance before and after weighting using standardized mean differences, with values < 0.1 indicating negligible imbalance and 0.1–0.2 suggesting minimal imbalance. We did not include POCUS as a variable in the propensity score model, as it was an outcome of interest rather than a baseline characteristic.

We analyzed group differences in LOS and cost using linear regression models within the IPTW-adjusted cohort. Results are presented as odds ratios (OR) or beta coefficients, each with 95% confidence intervals. All statistical analyses were performed using SAS software v9.4 (SAS Institute Inc., Cary, NC). Two-sided *P*-values < .05 were considered statistically significant.[Table t1-wjem-26-1564]

## RESULTS

### Baseline Demographics

**Figure 1:**
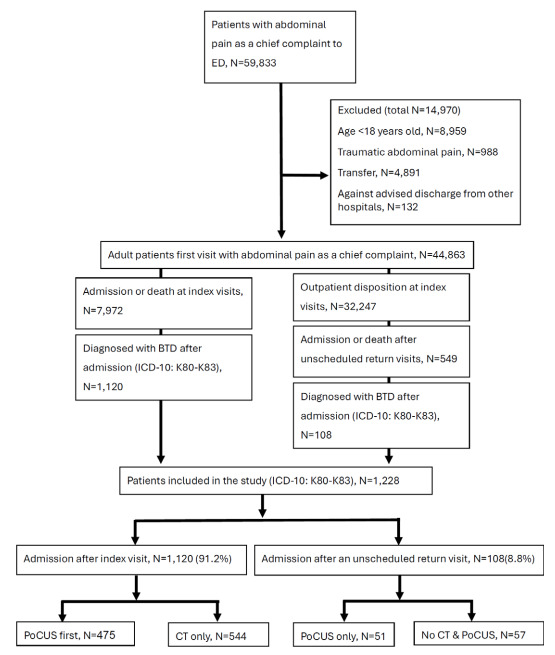
Flow diagram of study population selection. *ED*, emergency department; *BTD*, biliary tract disease; *POCUS*, point-of-care ultrasound; *CT*, computed tomography.

A total of 1,228 adult patients admitted with the diagnosis of biliary tract disease were included in the study, comprising 1,120 (91.2%) patients admitted during the index ED visit and 108 (8.8%) patients admitted after an unscheduled return visit within 72 hours (Figure 1).

After IPTW adjustment, baseline characteristics between groups achieved acceptable balance (standardized mean differences < 0.1). Even after adjustment, patients admitted after an unscheduled return visit had significantly more POCUS and fewer CTs at the initial visit. Before adjustment, patients admitted after an unscheduled return visit were younger (53.5 vs 59.2 years, *P* < .001), more likely to be male (62.0% vs 52.1%, *P* = .04), and had a higher BMI, lower severity of illness at triage, more POCUS, and less CT ordered.

### Quality of Care

As shown in Table 2, after IPTW adjustment, patients admitted after an unscheduled return visit had a significantly shorter initial ED LOS compared to those admitted during the index visit (*P* < .001). The ED LOS was reduced by approximately 400 minutes. ED costs at the index visit were also lower in the unscheduled return visit admission group (*P* < .001). The ED costs represented a saving of approximately 66%. No significant differences were observed between groups in total hospitalization costs, hospital LOS, ICU admission, or in-hospital mortality. This suggests that even though patients admitted after an unscheduled return visit had a shorter initial ED LOS and lower costs, patient safety was not compromised.

### Safety of Point-of-care Ultrasound in Patients Admitted after Unscheduled Return Visit

As shown in Table 3, the POCUS-guided discharge at the index visits in patients admitted for biliary tract disease after an unscheduled return visit exhibited a significantly shorter initial ED LOS (*P* < .001) and lower initial ED costs (*P* < .001) compared with patients who underwent CT and were admitted directly at the index visit. No significant differences were observed in admission costs, total costs, hospital LOS, or ICU LOS. As only one patient (0.9%, 95% CI, 0.02–5.1%) who received POCUS alone at the index visit and was admitted to the ICU for two days after an unscheduled return visit and no patients died (0%, 95% CI, 0–2.78 %), the ICU admission OR and in-hospital mortality OR could not be estimated due to the low event rate, suggesting that the use of POCUS did not compromise patient safety.

### Cost and Efficiency of Ultrasound in Patients Admitted After an Unscheduled Return Visit

As shown in Table 4, among patients admitted for biliary tract disease after an unscheduled return visit, those who received POCUS during the index visit (n = 51) had significantly shorter initial ED LOS (*P* =.006) compared with those who did not receive POCUS or CT (n = 57). The ED LOS was reduced by approximately 85 minutes. The initial ED costs were also significantly lower in the POCUS group (NT$3,248 vs NT$7,149; *P* < .001). The ED costs represented a saving of approximately 55%. However, no significant differences were observed in hospital LOS, admission costs, or total healthcare costs. These results highlight POCUS as a cost-efficient diagnostic tool that reduces initial ED resource use without adversely affecting patient outcomes.

## DISCUSSION

This study evaluated the association of POCUS with resource utilization and safety outcomes in patients admitted for biliary tract disease after an unscheduled return visit. A key finding is that admission did not correlate with worse clinical outcomes or increased overall costs compared to patients admitted during their index visit. This suggests that initial discharge decisions, facilitated by standard ED assessment, potentially augmented by POCUS, effectively identify many low-risk patients without compromising safety. Furthermore, our results indicate that POCUS-guided management during the initial ED encounter was associated with reduced ED LOS and initial ED costs, without adversely affecting patient safety metrics upon subsequent admission. These findings support the potential of POCUS as a valuable tool for risk stratification and resource optimization in the evaluation of adult patients presenting with non-traumatic abdominal pain.

### Outcomes of Patients Admitted After Unscheduled Return Visit

While delays in surgical intervention for acute biliary conditions can lead to complications,^33^ our cohort of patients admitted for biliary tract disease after an unscheduled return visit did not demonstrate increased overall hospital LOS, costs, ICU admission rates, or mortality compared to those admitted directly. These patients included those who were initially diagnosed with biliary tract disease and discharged for outpatient follow-up, and those who first presented with nonspecific abdominal pain and were later diagnosed with biliary tract disease on return. These scenarios reflect the diagnostic complexity of biliary tract disease in the ED. Patients discharged after an initial diagnosis for biliary tract disease often lack signs of acute inflammation or obstruction, making outpatient management appropriate. Conversely, patients with initially vague symptoms may only be diagnosed with biliary tract disease upon re-presentation, underscoring the condition’s potential to progress or evolve diagnostically over time.

Including both patient types provides a comprehensive view of how POCUS can support early identification and risk stratification of biliary tract disease, even when symptoms are atypical or biliary tract disease is not initially suspected. Our findings suggest that POCUS can enhance diagnostic accuracy across the full spectrum of ED presentations, reinforcing the value of the initial evaluation in identifying patients suitable for non-emergent outpatient care.

Importantly, unplanned return visits are not always avoidable. A study in New York State revealed that 48.6% of patients with biliary colic may not require surgery within five years, and one-third undergo cholecystectomy elsewhere.^34^ This demonstrates that the disease may progress and that we cannot avoid all unplanned return visits. Patient loyalty to our ED for return visits may indicate trust in care quality. Although unscheduled return visits. can occur, potentially due to disease progression^35^ or patient factors,^34^ our data suggest that these return admissions for biliary tract disease are not necessarily indicative of substandard initial ED care, a point supported by literature showing ED care quality is often not the primary driver for return admissions.^36^ Hospital management should avoid penalizing EDs for biliary tract disease readmissions, as these are not reliable quality metrics. Ensuring timely outpatient surgical follow-up remains a crucial component of managing symptomatic biliary colic effectively.^37^

### Point-of-care ultrasound and Emergency Department Efficiency

Consistent with prior research, our findings suggest that POCUS enhances ED efficiency in the context of suspected biliary tract disease.^15^, ^38^ By providing real-time imaging, POCUS expedites gallstone detection (a reliable application, although operator-dependent).^38^ Common errors, such as misinterpreting artifacts or failing to visualize the gallbladder neck,^27^ underscore the need for ongoing training. In the past, clinicians could order an ultrasound performed by a radiologist to aid in the diagnosis. However, this is time-consuming, and recent evidence suggests that obtaining an ultrasound by a radiologist after a positive POCUS by a qualified emergency physician requires additional time and may increase diagnostic uncertainty.^39^ Targeted ultrasound education can further optimize ED workflow, but clinicians must recognize POCUS limitations, including false negatives in conditions like appendicitis or challenges in obese patients. Clear protocols for when to pursue adjunctive imaging (eg, CT) are essential to ensure diagnostic accuracy.

### Point-of-care Ultrasound as a Decision-Making Tool

Abdominal pain poses a diagnostic challenge in the ED due to its diverse etiologies. Point-of-care ultrasound augments clinical assessment with immediate bedside imaging, facilitating rapid differentiation of patients requiring hospitalization vs discharge.^40^–^42^ This immediate imaging capability facilitates the timely differentiation of patients requiring advanced diagnostics or hospitalization and holds potential for reducing healthcare expenditures.^43^ Existing evidence underscores the diagnostic accuracy of POCUS, demonstrating comparability to standard imaging for identifying critical conditions such as aortic aneurysm and gallbladder pathology.^44^ Furthermore, the integration of POCUS into the diagnostic workflow is associated with improved clinical decision-making by significantly narrowing the differential diagnosis, guiding management strategies, and reducing the need for ancillary testing.^45^ The POCUS findings directly informed timely disposition decisions, reduced diagnostic uncertainty, and improved workflow. However, POCUS must complement a comprehensive evaluation, including history, physical examination, and laboratory results. Evidence-based guidelines are needed to standardize POCUS indications and clarify when advanced imaging is warranted, ensuring accurate and timely diagnoses.

### Point-of-care Ultrasound and Emergency Department Costs

Point-of-care ultrasound offers potential economic advantages in the ED by mitigating the need for resource-intensive advanced imaging.^46^,^47^ Studies demonstrate its potential, such as reducing subsequent CT utilization for abdominal pain,^48^ and POCUS demonstrates diagnostic advantages over radiography for certain conditions like small bowel obstruction, potentially replacing CT or MRI in select cases.^49^ These shifts in diagnostic pathways can decrease direct imaging costs. In this study, almost no patients admitted for biliary tract disease at our hospital were managed with POCUS alone. Therefore, we did not compare the cost and efficiency of POCUS in patients admitted directly for biliary tract disease at the index visit. However, focusing on patients admitted for biliary tract disease after an unscheduled return visit, we found that index-visit management using POCUS alone was associated with lower initial ED LOS and costs compared to patients not receiving index-visit POCUS. This observed reduction in LOS aligns with literature suggesting POCUS can shorten ED LOS and optimize resource utilization, potentially reducing demand on beds, staff, and supplies.^50^

It is also important to consider that physicians comfortable and willing to perform POCUS may inherently be more proactive in their diagnostic approach, a factor that could contribute to the observed reductions in LOS and resource utilization.^51^, ^52^ When used selectively in appropriate clinical scenarios, POCUS offers meaningful opportunities for both cost savings and improved care delivery for patients with abdominal pain, without compromising patient safety. These results support health policy initiatives that expand POCUS training and certification for emergency physicians. Due to its portability, affordability, and versatility, POCUS is well-suited for scalable adoption across a range of ED environments, including rural and resource-limited settings. Standardizing POCUS use and incorporating it into national quality metrics could further strengthen diagnostic pathways and optimize emergency care.

## LIMITATIONS

This study has several limitations. First, as a retrospective cohort study, it is subject to selection bias and unmeasured confounders. Despite statistical adjustments, residual confounding may still affect the results. Certain clinical details, such as specific symptoms, patient flow, and physicians’ discretion in selecting POCUS or CT, were not documented. Second, this study was conducted at a single, tertiary medical center in Taiwan, which may limit its generalizability to other healthcare settings, particularly non-Asian or resource-limited EDs with different POCUS training standards, protocols, resources, and patient demographics. The study population included patients with more severe comorbidities, and a higher proportion of those with malignancies underwent CT-only evaluation, potentially influencing imaging choices and outcomes.

Third, physician-related factors, such as variation in diagnostic interpretation and POCUS expertise, were not captured and may have influenced outcomes. As an operator-dependent tool, the effectiveness of POCUS is closely tied to clinician experience and training.^53^ This study did not distinguish between POCUS performed by highly experienced physicians and that performed by less experienced users, potentially introducing variability. Additionally, the choice of diagnostic imaging could have been influenced by individual physicians’ familiarity with POCUS or a prevailing preference for CT, which some clinicians consider more reliable for evaluating abdominal pain. Fourth, we did not assess the diagnostic accuracy of POCUS or the impact of training level on clinical decision-making. However, all POCUS examinations and discharge decisions by residents were supervised by attending physicians, which likely mitigated variability. Moreover, attending physicians retained full discretion to order confirmatory CT imaging when clinical uncertainty remained.

Fifth, we did not exclude patients with a known history of biliary tract disease, which may have influenced the decision to perform POCUS. For instance, in patients with prior interventions such as percutaneous transhepatic cholangial drainage or gallbladder drainage, clinicians may prefer laboratory testing or abdominal CT. In contrast, POCUS remains a viable first-line imaging modality in patients with a history of cholelithiasis or cholecystectomy. Including these patients reflects routine ED practice and enhances the generalizability of our findings on POCUS use in abdominal pain. Sixth, our definition of the index visit, no prior ED visit or hospitalization within 72 hours, aligns with standard definitions of unscheduled return visits in ED. While this short interval may allow for some confounding from recent encounters, it is widely used in studies focused on potentially avoidable, short-term revisits. A longer washout period (eg, 30–60 days) might reduce this risk but could also limit the generalizability of findings by excluding patients with unrelated prior care.^54^,^55^

Seventh, we analyzed only the first return visit within 72 hours for each patient during the study period. This approach ensures analytic consistency but may underrepresent patients with multiple revisits, who could signal a higher-risk subgroup. Nonetheless, the first return visit is generally more indicative of potentially preventable issues directly related to the initial ED evaluation. Subsequent revisits tend to reflect more complex or evolving conditions, less influenced by initial decision-making. Eighth, our dataset lacked detailed clinical variables such as lab results, imaging findings, and symptom severity scores, limiting our ability to fully explain revisit causes or isolate the causal effect of POCUS. To address this limitation, we applied propensity score weighting to balance baseline characteristics between groups, achieving standardized mean differences < 0.1.

Ninth, because our study focused on patients with biliary tract disease, inclusion of POCUS exams for non-biliary indications could introduce bias. However, in the ED setting, POCUS is typically used in a broad, question-driven manner without rigid protocols.^56^ This reflects real-world diagnostic workflows and supports the generalizability of our findings. Lastly, there was an imbalance in the number and severity of patients between groups, which may have introduced selection bias. This discrepancy reflects real-world practice, where emergency physicians often have individual preferences for using ultrasound or CT during evaluations. Implementing a randomized study design could help eliminate this bias and provide a more accurate assessment of the clinical efficacy of POCUS and CT.

## CONCLUSION

Our analysis revealed two key findings: First, patients admitted for biliary tract disease following an unscheduled return visit had lower resource utilization during their initial ED visit without apparent safety compromise. Second, implementing discharge decisions for undifferentiated non-traumatic abdominal pain guided by point-of-care ultrasound significantly reduced ED length of stay and costs without increasing adverse outcomes or total hospital costs, even among those later admitted for biliary tract disease. These results imply that current ED assessment strategies, enhanced by POCUS, can effectively risk-stratify patients, improving departmental efficiency and value.

Point-of-care ultrasound appears particularly useful for bolstering diagnostic confidence and expediting disposition decisions. However, widespread adoption requires targeted emergency physician training and robust guidelines. Prospective, multicenter studies are warranted to validate these findings across diverse emergency settings and patient populations and to further define the role of POCUS in optimizing care for patients with suspected biliary tract disease.

## Figures and Tables

**Table 1 t1-wjem-26-1564:** Demographics of the study population on quality of care in patients with biliary tract disease.

	Before IPTW			After IPTW		
	
	Admission after index visit, N = 1,120	Admission after an unscheduled return visit n = 108	*P*-value	Admission after index visit (%)	Admission after an unscheduled return visit (%)	SMD
Age	59.2 ± 17.0	53.5 ± 15.1	< .001	58.7 ± 17.8	58.3 ± 55.7	0.008
Sex			.04			
Male	583(52.1)	67(62.0)		52.9	49.5	−0.094
Female	537(48.0)	41(38.0)		47.1	50.5	0.098
BMI	25.2 ± 4.6	26.0 ± 3.9	.04	25.3 ± 4.9	25.7 ± 12.5	−0.038
Triage			.006			
1	16(1.4)	0(0.0)		1.3	0.0	−3.866
2	268(23.9)	12(11.1)		22.8	24.4	0.095
3	820(73.2)	95(88.0)		74.5	74.6	0.001
4	16(1.4)	1(0.9)		1.4	1.1	−0.869
5	0(0.0)	0(0.0)		0.0	0.0	-
Heart rate	90.0 ± 19.9	78.6 ± 13.5	< .001	89.9 ± 20.8	78.2 ± 44.2	0.338
Systolic blood pressure	136.6 ± 25.3	144.4± 27.7	.002	136.6 ± 26.4	145.2 ± 91.8	−0.126
Diastolic blood pressure	80.5 ± 14.2	84.8 ± 15.2	.003	80.7 ± 14.9	83.2 ± 47.6	−0.071
Body temperature (^o^C)	36.9 ± 1.0	36.4 ± 0.6	< .001	36.9 ± 1.0	36.5 ± 2.1	0.267
Respiratory rate	19.6±1.6	19.5 ± 1.4	.39	19.6 ± 1.7	19.5 ± 4.7	0.030
History of comorbidities	774(69.1)	69(63.9)	.26	68.6	68.5	−0.002
POCUS	475(42.4)	71(65.7)	<0.001	42.6	64.9	0.594
CT	960(85.7)	29(26.9)	<0.001	85.7	31.3	−1.326

This table presents the baseline characteristics of patients included in the study examining the quality of care for biliary tract disease in the emergency department, comparing those who received POCUS to those who did not. After IPTW adjustment, the groups achieved acceptable covariate balance, defined as SMD < 0.1. Before adjustment, patients who were admitted after an unscheduled return visit were generally younger, more often male, and had a higher BMI, lower triage-assessed severity of illness, higher rates of POCUS use, and lower rates of CT ordering. Data are presented as frequency (percentage) or mean ± SD.

*BMI*, body mass index; *CT*, computed tomography; *IPTW*, inverse probability of treatment weighting; *LOS*, length of stay; *SMD*, standardized mean difference; *POCUS*, point-of-care ultrasound.

**Table 2:** Quality of care in patients admitted for biliary tract disease after an unscheduled return visit.

Outcome measures, point estimate (95% CI)	Admission after the index visit	After IPTW	

		Admission after an URV	*P*-value
Initial ED LOS (hours)	Ref.	−6.66 (−8.60 to −4.71)	< .001
Initial ED costs (NT$)	Ref.	−5,647.9 (−7,260.1 to −4,035.8)	< .001
Total costs (NT$)	Ref.	−5,078.8 (−15,083.9 to 4,926.4)	.31
Hospital LOS (day)	Ref.	−1.12 (−2.41 to 0.17)	.08
ICU, OR	Ref.	1.78 (0.19 to 16.45)	.61
Expired in ED, OR	Ref.	0.18 (0.02 to 2.12)	.17
Expired after admission, OR	Ref.	0.59 (0.10 to 3.45)	.56

After adjustment using IPTW, the analysis showed that patient safety was not compromised, despite patients having a shorter LOS during the initial ED visit and incurring lower associated costs. Results were adjusted for age, sex, triage level, BMI, and comorbidities.

*BMI*, body mass index; *CT*, computed tomography; *ED*, emergency department; *ICU*, intensive care unit; *IPTW*, inverse probability of treatment weighting; *LOS, length of stay*; *OR*, odds ratio; *NT$*, new Taiwan dollar; *Ref.*, reference.

**Table 3:** The safety of ultrasound-guided discharge at the index visits in patients admitted for biliary tract disease after an unscheduled return visit.

Outcome measures, point estimate (95% CI)	Admission after index visit with CT N = 544	After IPTW	

		Admission after an unscheduled return visit with POCUS only N =. 51	*P*-value
Initial ED LOS (hours)	Ref.	−13.71 (−15.19 to −12.22)	< .001
Initial ED costs (NT$)	Ref.	−13,566.8 (−14,907.2 to −12,226.4)	< .001
Admission costs (NT$)	Ref.	−12,938.2 (−28,793.2 to 2,916.9)	.10
Total cost (NT$)	Ref.	−8,553.2 (−25,851.6 to 8,745.2)	.33
Hospital LOS (day)	Ref.	−1.45 (−4.20 to 1.30)	.30
ICU LOS (day)	Ref.	−2.25 (−5.45 to 0.96)	.15
Expire, OR	Ref.		

This table presents patient safety outcomes in the study evaluating the use of POCUS at the index visit among patients admitted for biliary tract disease after an unscheduled return visit to the ED. After adjustment using IPTW, POCUS-guided discharge at the index visit, with subsequent admission after an unscheduled return visit, was not associated with compromised patient safety when compared with patients who underwent computed tomography and were admitted directly at the index visit. Analyses were adjusted for age, gender, triage level, body mass index, and comorbidities.

*ED*, emergency department; *ICU*, intensive care unit; *IPTW*, inverse probability of treatment weighting; *LOS*, length of stay; *NT$*, new Taiwan dollar; *OR*, odds ratio; *POCUS*, point-of-care ultrasound; *Ref.*, reference.

**Table 4:** The association of cost and efficiency and point-of-care ultrasound in patients admitted for biliary tract disease after an unscheduled return visit.

Outcome measures, point estimate (95% CI)	Admission after an unscheduled return vist without CT and POCUS, N = 57	After IPTW	

		Admission after an unscheduled return visit visit with POCUS only, N = 51	*P*-value
Initial ED LOS (hours)	Ref.	−1.42(−2.44 to −0.41)	.006
2nd ED LOS (hours)	Ref.	−1.08(−6.98 to 4.82)	.71
Total LOS in ED (hours)	Ref.	−2.51(−8.31 to 3.29)	.39
Initial ED costs (NT$)	Ref.	−3,270.7(−4,505.1 to −2,036.3)	< .001
2nd ED costs (NT$)	Ref.	2,026.9(−3,058.7 to 7,112.7)	.43
Admission costs (NT$)	Ref.	−1,370.1(−20,661.9 to 17,921.6)	.88
Total costs (NT$)	Ref.	−4,675.4(−26,028.8 to 16,677.9)	.66
Hospital LOS	Ref.	−0.07(−3.19 to 3.06)	.96

This table presents findings from the study evaluating the association of cost and efficiency and POCUS use during the index ED visit in patients who were later admitted for biliary tract disease following an unscheduled return visit. After IPTW adjustment, patients who received POCUS during the initial ED visit had significantly lower ED LOS and reduced initial ED costs compared with those who did not receive POCUS and computed tomography. Analyses were adjusted for age, sex, triage level, body mass index, and comorbidities.

*ED*, emergency department; *ICU*, intensive care unit; *IPTW*, inverse probability of treatment weighting; *LOS*, length of stay; *OR*, odds ratio *NT$*, new Taiwan dollar; POCUS, point-of-care ultrasound; *Ref.*, reference.
